# Analysis of the Effect of Layer Height on the Interlayer Bond in Self-Compacting Concrete Mix in Slab Elements

**DOI:** 10.3390/ma17164005

**Published:** 2024-08-12

**Authors:** Piotr Dybeł, Milena Kucharska

**Affiliations:** Faculty of Civil Engineering and Resource Management, AGH University of Krakow, Al. A. Mickiewicza 30, 30-059 Krakow, Poland; kucharska@agh.edu.pl

**Keywords:** multilayer casting, X-ray computed tomography, self-compacting concrete, interlayer bond strength

## Abstract

This paper presents a study on the influence of the layered casting technology of self-compacting concrete (SCC) on the load-bearing capacity of interlayer bond in slab elements. The research was conducted on slab elements with dimensions of 750 × 750 × 150 mm, concreted from a single point of concrete delivery. The aim of this study was to analyse the influence of the height of the concreting top layer on the bond strength between the layers. The study utilised top layer heights of 50, 75, and 100 mm, which, according to the authors’ experience, are the most common cases when making slab elements. The interlayer bond was determined by investigating the splitting tensile strength of cubic specimens cut from the concrete slabs. Computed tomography (CT) was employed to image the contact zone between the concrete layers. Based on the analysis of the CT imaging and the results of the strength tests, it was shown that the interlayer bond is influenced by both the height of the top layer and its free-spread distance from the casting point. A reduction in the interlayer bond strength was observed with decreasing the height of the top layer and increasing distance from the mixture supply point. The relationships obtained were linear and had a clearly negative slope. It was concluded that the valid recommendations and standards for the multilayer casting of SCC are too general. Therefore, we propose to detail the recommendations to reduce the risk of cold joints, which diminish the bond strength of the interlayer joints

## 1. Introduction

Self-compacting concrete (SCC) is now widely used in monolithic construction. Due to its superior working performance, self-compacting concrete is the material of choice for structures requiring greater durability and resistance to aggressive environments, such as bridges, marine structures, and tunnel casings [1,2,3]. The increased fluidity of SCC is due to its special composition, which includes a high proportion of fine-grained fillers such as fly ash. This property allows SCC to be used to cast concrete in complex shapes and in areas of dense reinforcement, where traditional concretes may have difficulty in fully filling the spaces [4,5,6]. A significant challenge when concreting reinforced concrete structures is ensuring that batches of concrete placed at different times are integrated. This situation arises when concreting in layers and during technological breaks. The interface between the layer of mix, in which certain physical and mechanical processes have already taken place, and the layer of fresh concrete mix is a potential zone of discontinuity in the concrete [7,8,9]. Furthermore, they are the weakest part of the construction and therefore a hidden fault. In addition, a permeable layer is created in this zone, which raises durability issues. In the case of reinforced concrete, the interaction between concrete and steel can also be impaired if the interface between the layers is close to the reinforcement [10]. Furthermore, the application of the concrete mix may result in the formation of unsightly lines and discolouration on the external surfaces due to interruption of the placement process.

The issues associated with layered construction originate from the stiffening effect of a self-compacting mix at rest, which is related to the thixotropic change in viscosity [8,11]. According to [12], this phenomenon can occur within a few minutes. When casting concrete in multiple layers, there is a risk that the following layer of mix will be placed on top of a layer that has already begun to stiffen. Consequently, inadequate mixing of the subsequent layers of concrete and a reduction in interlayer bond strength are anticipated. Therefore, technological solutions are sought to enhance the integration of successive layers of SCC mix. Some general technological recommendations are included in the standard guidelines [13,14,15]. However, there is currently no method for unambiguously defining the operating procedures for the execution of multilayer concreting using SCC. Recommendations are based on contractor experience and/or preliminary tests [16]. Currently, there are no simple tests that can be used to determine the ability of SCC mixtures to be multilayered on site.

A study of the multilayered concreting of slab elements has revealed that three key factors influence the decrease in the bond strength between layers. These are the delay time, the distance from the mixture casting point, and the method of first layer preparation [17]. The greatest reduction in interlayer bond strength was observed when the mix flowed freely onto the previously laid layer. The reduction in load-bearing capacity for a delay of 60 min reached as much as 28.4%. Conversely, when the previously laid layer was mechanically disturbed, the decrease in load-bearing capacity was 11.4%. Similar results were reported in experiments on beam elements [18]. A decrease in mechanical strength of more than 40% was observed in specimens with cylindrical cores subjected to a simple compression test [12]. The authors proposed that issues in casting separate layers can only arise in the event of a smooth interface between two layers [19]. At long delay times (more than 60 min), the influence of base roughness increases, which increases the bond strength due to greater mechanical interlocking [20]. A study [8] demonstrated that even if the two layers do not mix during the casting of the top layer, the surface roughness of the bottom layer can be sufficient to ensure that the final mechanical strength is not reduced by casting separate layers. The surface roughness of the fresh concrete in the bottom layer, for example, can result from the uplift of coarse aggregate as a result of ensuring proper mix composition and stability. It was also found that the critical delay time between casting layers depends largely on the thixotropic properties of the SCC, the thickness of the layers, and the roughness of the interface between the layers. The authors recommended that the SCC should be as thixotropic as possible when casting the slabs, with a low structuring ratio (e.g., less than 0.1 Pa/s). Furthermore, it has been demonstrated that even in the case of the most viscous SCC, shear stresses at the interface can be neglected due to the influence of the weight of the second layer once its height exceeds 100 mm. In the case of layers with a significant height, spontaneous mixing of the mix layers occurs due to the influence of their weight. One promising approach to reduce the effect of structural build-up at rest during multilayer casting is the use of polymeric bonding agents on the surface of the first layer [21]. Research is currently being conducted to establish the critical delay time, defined as the longest time after which the residual bond strength is at least 90% of the strength reached during continuous SCC placement [22,23].

The issue addressed in this paper is of great importance for the technology of multilayer concreting of elements using self-compacting concrete. The objective of this study is to analyse the influence of the concrete layer height of slab elements for the case of a smooth interface between two layers on the interlayer bond strength. The analysis was carried out to determine the interlayer bond strength of the SCC layers when a top layer height of up to 100 mm is used. In the authors’ experience, this is the most commonly encountered scenario during the execution of the slab elements. It is of paramount importance to ensure an adequate flow of the successive SCC layer after the previously placed one, in order to guarantee the high quality and durability of the final concrete structure. In this study, strength tests as well as X-ray computed tomography (CT) were employed in order to image the contact zone of the concrete layers.

## 2. Materials and Methods

### 2.1. Concrete Mixture

The SCC mix utilised in the study was based on blast furnace cement CEM III/A 42.5 N, with a dosage of 450 kg/m^3^. The binder met the requirements of EN 197 [24]. The water-cement ratio of the mix was 0.36. Three different aggregate fractions were employed: 685 kg/m^3^ of natural sand with a particle size ranging within 0 ÷ 2 mm, 510 kg/m^3^ of gravel aggregate with a particle size ranging within 2 ÷ 8 mm, and 560 kg/m^3^ of gravel aggregate with a particle size ranging within 8 ÷ 16 mm. The aggregates met the requirements of EN 12620 [25]. Chemical admixtures included a superplasticiser in the form of polycarboxylic ether polymer at 4.95 kg/m^3^ and a plasticiser at 2.25 kg/m^3^.

### 2.2. Specimens and Basic Modules

**Figure 1:**
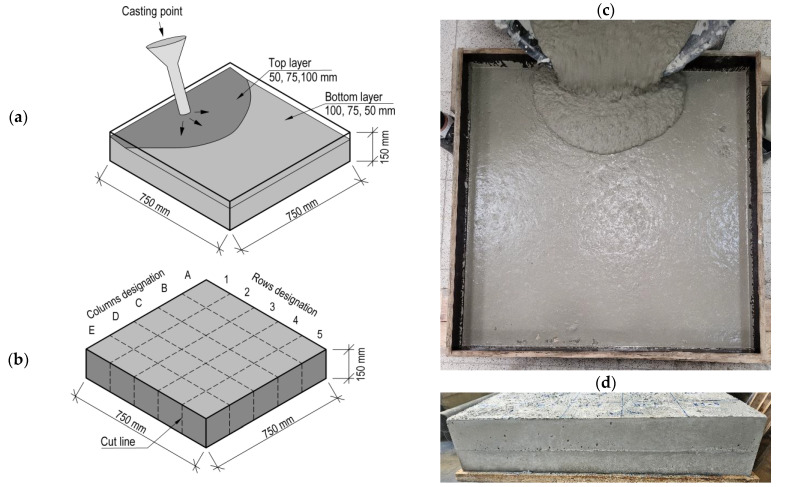
Test pieces used in the study: (**a**) scheme of layered casting of slabs; (**b**) identification of specimens; (**c**) realisation of the top layer execution; (**d**) example of a connection line.

A slab element with dimensions of 750 mm in length, 750 mm in width, and 150 mm in height (Figure 1) was employed in the experimental study. The design of the element was based on cube base modules with dimensions of approximately 150 mm length, 150 mm width, and 150 mm height. The construction of the element was carried out in two stages. In the initial stage, a layer of SCC was cast to a height of 50, 75, or 100 mm. In the subsequent stage, the forms were completed to a full height (150 mm). The simulation of concrete casting technology for slabs and interruptions in the delivery schedule was conducted by extending the execution time for the second stage. Based on the findings of previous studies [17], an interval of 60 min was assumed for the placement of the subsequent layer of mix. At shorter delay times, a slight deterioration in the interlayer bond strength of the joint was observed. No treatments were employed to enhance the bond forces between the layers. The mixes in each variant were delivered from a fixed casting point located at one end of the form, which was intended to simulate the free flow of the SCC mix along the formwork.

The elements were maintained in a fixed position under laboratory conditions throughout the concrete curing period. Curing and protection of the concrete were conducted by water sprinkling and covering it with plastic sheets during its early life. The elements were precisely cut into elementary parts with a concrete saw 21 days after concreting. One slab element was made for each test series, resulting in a total of 25 base specimens of the respective model. Consequently, a total of 75 specimens were prepared for the study. In addition, 10 cubic specimens measuring 150 mm × 150 mm × 150 mm were prepared in a single layer. These were used for compressive strength and splitting tensile strength testing. Two concrete cores were extracted from each slab for examination of the interlayer contact using X-ray computed tomography. One specimen was located in the zone of the concrete mix discharge (designated C-1), while the other was situated in the zone on the opposite side of the discharge location (designated C-5).

### 2.3. Test Procedures

A series of three tests were conducted to assess the properties of the fresh concrete mix. The first of these was the flow test, which enabled the flow and plastic viscosity of the concrete to be determined [26]. The second test was the L-box test, which assessed the capacity of the concrete mix to flow freely through tight openings [27]. The third test was a visual assessment of the segregation resistance of the SCC mix [28]. Testing of the fresh concrete mix was carried out directly prior to its placement in the mould.

**Figure 2:**
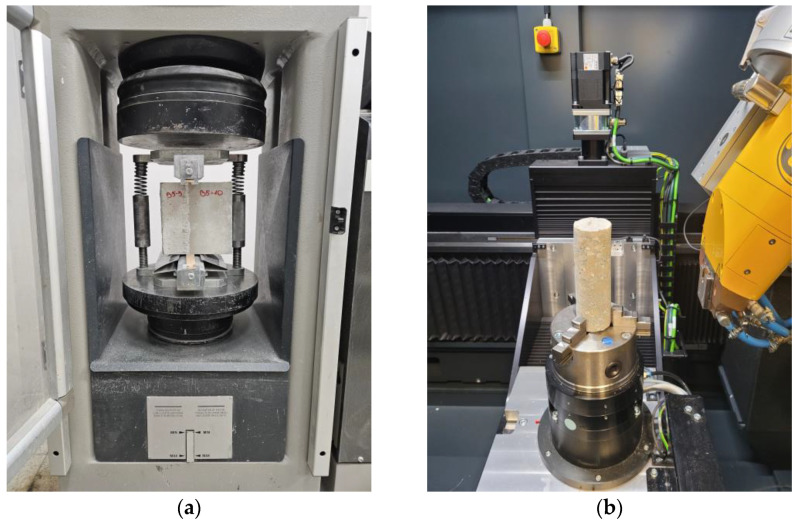
Performance of tests: (**a**) interlayer bond strength; (**b**) X-ray computed tomography.

The compressive strength of the concrete was evaluated in accordance with the specifications outlined in EN 12390-3 [29]. The splitting tensile strength test was conducted in accordance with the procedure set forth in EN 12390-6 [30]. The interlayer bond strength of the SCC layers was evaluated using a splitting tensile test [30]. The contact between the two layers of the self-compacting mix was positioned vertically, in the axis of the acting forces (Figure 2a). The interface bond strength values were calculated in accordance with the procedure for determining the concrete splitting tensile strength.

X-ray computed tomography was used to analyse the interface between the layers (Figure 2b). X-ray computed tomography entails the reflection of a material’s three-dimensional structure from multiple planar tomographic views, employing X-ray spectroscopy. Following the image reconstruction process, any discrepancies in the material, such as changes in density or the presence of voids, can be visualised and quantified [31,32,33]. All samples were scanned at fixed scanning parameters using a GE Phoenix v|tomex|m tomograph (ITA, Poznan, Poland). The reconstruction process, which included combining a series of two-dimensional images into a three-dimensional model, was conducted using Volume Graphics VGSTUDIO MAX 2024.2 software. 

## 3. Results and Discussion

### 3.1. Fresh Mix Properties and Concrete Strength

The fresh mix properties were tested to determine the slump flow and slump flow time (T500), which were found to be 645 mm and 5 s, respectively. Additionally, the ability to flow through tight openings was assessed using the L-box ratio, which demonstrated an acceptable range of 0.85. A visual assessment of segregation resistance indicated that the mix achieved an index of 1. Furthermore, analysis of the flow properties of the mix demonstrated that it exhibited adequate stability and fluidity. The mixture maintained its properties even when subjected to a prolonged mixing time, which was a crucial factor in the ongoing interlayer bond strength studies. The obtained rheological properties are in accordance with the European requirements for SCC [34], confirming that the mixture met the high-quality and performance standards required in modern construction. The compressive strength obtained on cubic reference specimens after 28 days of curing was 73.03 MPa, with a standard deviation of 4.33 MPa. The compressive strength of SCC was determined using 11 cubic specimens, each with a side of 150 mm.

### 3.2. Interlayer Bond Strength

**Figure 3:**
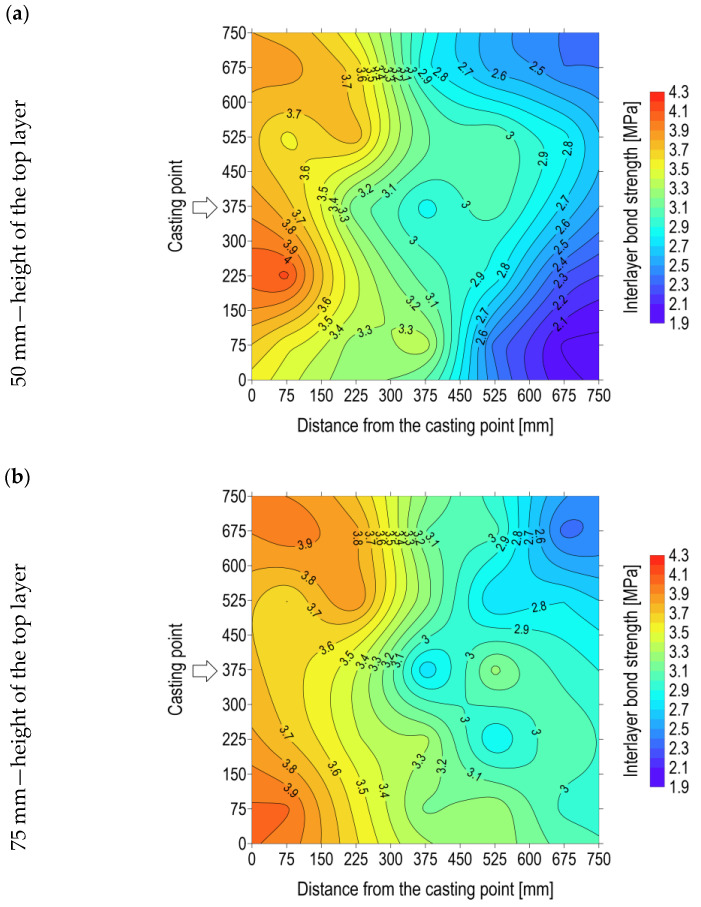

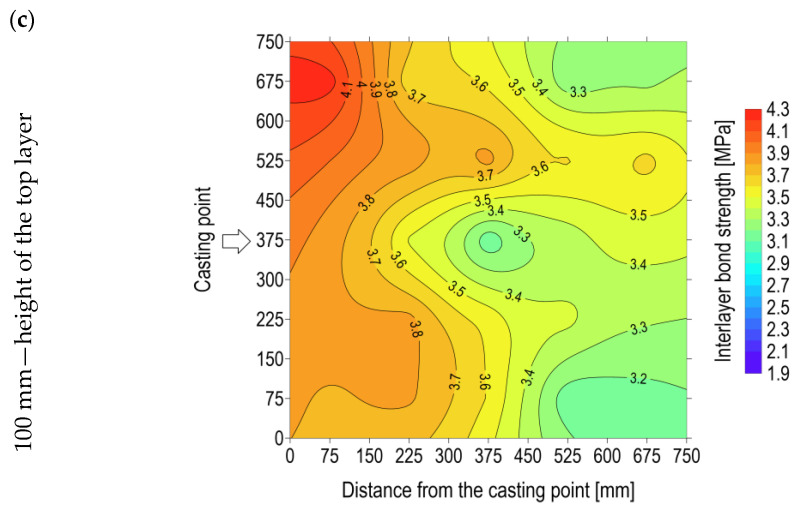
Distribution map of interlayer bond strength in a slab element: (**a**) height of the top layer 50 mm; (**b**) height of the top layer 75 mm; (**c**) height of the top layer 100 mm.

Table 1 presents the mean values of the interlayer bond strength for a given row, obtained through the analysis of specimens extracted from slab elements. The mean value for rows 1 and 5 was determined based on four specimen test results, while for the remaining rows, it was determined based on five specimen test results. This was due to the designation of one sample from each of rows 1 and 5 for X-ray examination (samples C-1 and C-5). In order to facilitate the interpretation of the results, an approximate map of the distribution of the interlayer bond strength was produced in the form of a colour map and isolines (Figure 3). The interlayer bond strength outcomes for each specimen, derived from the slab (a total of 23 specimens out of 25 for each element), were associated with the location coordinates corresponding to the centre of the specimen. This resulted in the generation of a grid of points. Subsequently, the Krining estimation method was employed to create a map of the interlayer bond strength distribution. Kriging is a geostatistical method for estimating the value of a feature throughout the area under study. This method was employed to obtain the continuity of the distribution of the interlayer bond strength in locations C-1 and C-5, as well as in areas beyond the centres of the test specimens. The analysis of the distribution of the interlayer bond strength indicates the presence of a zone of good mixing of the bottom and top layers at the mix delivery location. This observation verifies the recommendations of the technology for the multilayer casting of SCC [13,14]. The recommendations propose that, in the event of interruptions during the concreting process, successive layers of SCC mix should be placed in a manner that ensures the surface liquefaction of the previous layer, thereby facilitating its integration with the newly placed layer. The desired result may be attained by means of surface mixing the mix or via a gentle vibration. Potential alternative solutions may be to increase the mix pressure in the pipeline, or alternatively to raise the height from which it is poured. Nevertheless, the extent of the zone of good mixing due to mixture delivery was contingent upon the height of the top layer. It can be observed that the greater the height of the top layer, the larger the area of good mixing (Figure 3).

**Figure 4:**
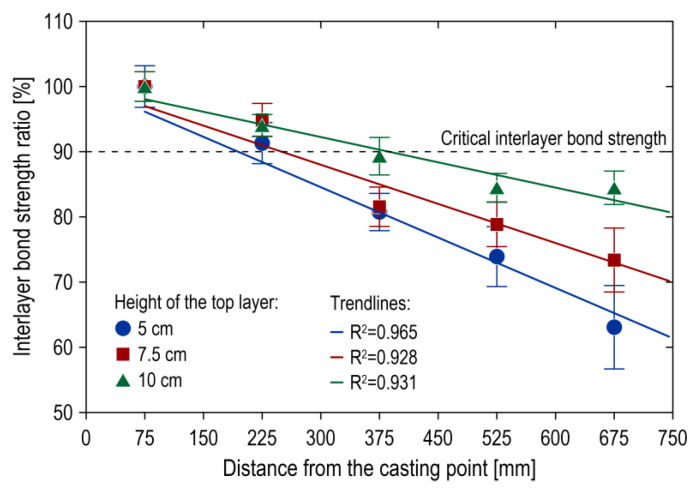
Relationship of the interlayer bond strength ratio to the distance from the casting point.

Figure 4 illustrates the variation in the interlayer bond strength ratio as a function of the distance of the specimens from the casting point. The interlayer bond strength ratio was defined as the proportion of the mean value obtained from the specimens in a given row (rows 2 to 5, respectively) to the mean value registered for the specimens in row 1 (the discharge location). The interlayer bond strength was observed to decrease with increasing distance from the casting point. The relationships observed were linear, with a clearly negative slope. It was demonstrated that the smaller the top layer, the more pronounced the impact of the distance of the specimen from the concreting area on the reduction in bond. For top layer heights of 50, 75, and 100 mm, the maximum decreases in the interlayer bond strength along the element relative to the interlayer bond strength at the casting point were 36.9%, 26.7%, and 15.4%, respectively. Furthermore, a greater degree of heterogeneity in the results was observed as the height of the top layer of the mix decreased. The findings of this study indicate that as the distance from the point of casting increased, the flowing mixture lost its velocity, becoming unable to generate sufficient shear stress to reverse the stiffening of the bottom layer structure. This effect was found to be significantly enhanced by the corner zones of the slab elements, where the lowest interlayer bond strength values were recorded. These zones were situated at the greatest distance from the point of mix discharge, and the presence of two formwork walls resulted in a reduction in the velocity of the flow due to friction against the formwork. When verifying the interlayer bond strength along the length of the element against the average value obtained on the monolithic specimens, a maximum decrease in interlayer bond strength was recorded. For a layer height of 50 mm, the maximum decrease was 38.2%, while for layer heights of 75 and 100 mm, the maximum decreases were 27.3% and 13.0%, respectively. 

**Figure 5:**
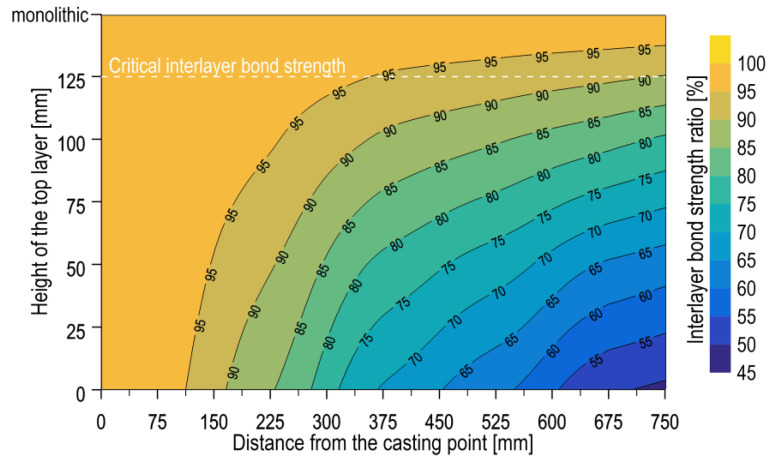
Interlayer bond strength in relation to the height of the top layer and the distance from the casting point.

Figure 5 presents an approximate map of the interlayer bond strength distribution in the form of a colour map and isolines, generated using the local regression method in Surfer software (Surfer 19.1.189). This approach permitted the estimation of interlayer bond strength for a wider range of top layer heights, with consideration of the distance of the interlayer joint from the concrete mix discharge. In the figure, a line was introduced to determine the residual bond strength of 90% of the average strength obtained on the monolithic specimens. It can be concluded from the aforementioned predictions that a top-layer height of more than 125 mm would be sufficient to achieve the necessary level of interlayer bond strength in the context of the present study. This is generally consistent with the recommendation in [9], although the value is strongly dependent on the distance from the casting point of the mix. In the event that the layer height is less than 125 mm, the free flow of the mix over the previously laid layer results in a reduction in the interlayer bond strength. It was demonstrated that the layer height was not the sole factor influencing the outcome; the distance from the mix casting point also played a role. As the distance from the concreting location increased, the mix lost its velocity and was unable to generate sufficient shear stresses at the boundary of the layers to reverse the stiffening of the lower layer structure. It follows that a single value cannot be adopted for the height of the upper layer in multilayer slabs casting to ensure the necessary interlayer bond strength is achieved. The results of the present research extend and complement the findings set out in the works [8,12] on the influence of the height of the top layer on the bond strength between the layers. 

**Figure 6:**
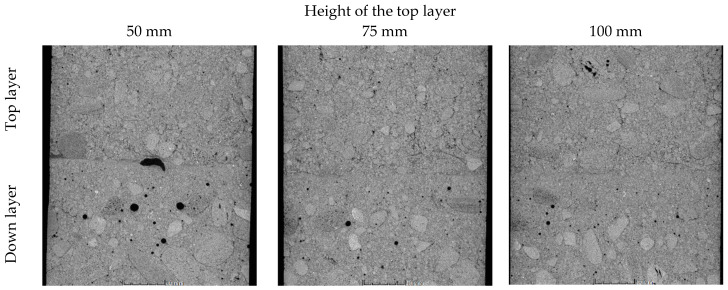
The X-ray computed tomography analysis images of the bottom and top layer interface for cores taken from the row on the opposite side from the casting point (samples C-5).

In order to elucidate the reasons for the observed reduction in bond strength between the two layers, an X-ray computed tomography imaging analysis of the interface between the layers was conducted. Cores from specimens located in the row of the mix discharge (specimens marked C-1) and in the row opposite the discharge (specimens marked C-5) were examined. The analysis of the C-1 cores did not reveal any changes in the concrete structure along the height of the specimens, suggesting that sufficient mixing of the concrete layers occurred at the point of casting, regardless of the height of the top layer. Beyond the region of mixture discharge, the top layer exhibited a free flow over the partially thickened bottom layer. The extent of mixing in the C-5 cores was dependent on the height of the top layer, with varying degrees observed (Figure 6). At a top layer height of 50 mm, a classic structural buildup at rest was evident at the contact zone. The joint produced was therefore similar in its structure to the joint between hardened concrete with a smooth surface and new concrete. An interfacial transition zone was observed at the interface between the layers, within which the failure plane passed. Increasing the height of the upper layer to 75 and 100 mm resulted in a reduction or disappearance of the interfacial transition zone. There was a mixing effect between the cement matrix and the fine aggregate in between the layers, but it was possible to approximate the course of the interface between the successive layers.

The prevailing international recommendations for the majority of structural elements advise the use of a single-point concrete placing technology until the formwork is completely filled. An alternative approach is to minimise the number of casting points and adapt them to the rheological properties of the SCC mix [13,14,15]. It is essential that the mix is able to spread over a sufficient distance in order to allow for adequate self-deaeration and self-levelling. This approach is effective for the placement of small elements or a continuous supply of mix. However, when concreting large-area slabs using the technology of uniform filling of the entire surface or when successive layers of mix are interrupted from one or more discharge points, this method is found to be unsuitable. The free flow of the mix over the initial layer does not guarantee adequate plasticization, particularly in the case of extended delay times. Furthermore, the height of subsequent spreading layers is typically insufficient to achieve the shear effect between the two layers, as demonstrated in this study to be crucial for improving the mixing of the two layers. For longer delay times (greater than 60 min), it is advisable to limit the free flow of the mixture over the previously laid layer, particularly in areas where the mixture loses its flow velocity, such as corners, narrowing of the formwork, and so forth. Alternatively, it is possible to use layers with an appropriate height, which should be adapted to the free flow distance. In the context of the test conditions presented in this paper, the top layer should be at least 125 mm high.

## 4. Conclusions

This article addresses the issue of the influence of the thickness of the overlying layer of concrete mixture on the bond between layers. The results of the strength and CT tests on samples with variants of layer thicknesses of 50, 75, and 100 mm have identified the following conclusions:

- In the context of the multilayer casting of slab elements, the delay in placing the top layer has a significant impact on the interlayer bond strength. It has been demonstrated that the interlayer bond strength is influenced by both the height of the top layer and its free-flow distance from the mix casting point.
- The study revealed that the lower the top layer, the more pronounced the impact of distance from the casting location on the decrease in interlayer bond strength. The maximum decrease in interlayer bond strength along the element compared to the monolithic specimen was observed for layers of 50, 75, and 100 mm, at 38.2%, 27.3%, and 13.0%, respectively.
- The study revealed significant differences in the mixing pattern of the concrete layers depending on the height of the top layer. The greater the height of the top layer, the more irregular the course of the layer contact and the more intense the mixing of the layers within the element cross-section. The mixing of the layers thus produced precludes the possibility of a continuous structural weakening of the structure.
- In the case of multilayer SCC placement, it is recommended that the free flow of the mix over the previously placed layer be restricted when concreting is interrupted for longer periods. This is particularly pertinent to areas where the flow speed is reduced, such as corners, narrowing in the formwork, and so forth. An alternative approach is to adjust the height of subsequent layers to align with the free-flow distance. In accordance with the test conditions presented in this study, the top layer should be at least 125 mm in height.

Research into the enhancement of the bond strength between concrete mix layers represents a pivotal area of investigation. The authors propose the expansion of research into the examination of diverse compositions of self-compacting mixes, placement speeds, and varying spread distances of the top layer depending on layer height. The objective is to ascertain in detail the influence of placement technology on the bond strength of concrete batches placed at different times.

## Figures and Tables

**Table 1:** The results of the average interlayer bond strength tests conducted on specimens extracted from slab elements.

Row Designation (See Figure 1)	Monolithic Sample		Height of the Top Layer					
			50 mm		75 mm		100 mm	
	f_ct,sp_ [MPa]	SD [MPa]	f_ct,sp_ [MPa]	SD [MPa]	f_ct,sp_ [MPa]	SD [MPa]	f_ct,sp_ [MPa]	SD [MPa]
1	3.85	0.40	3.77	0.24	3.82	0.17	3.96	0.18
2			3.44	0.24	3.62	0.20	3.73	0.14
3			3.04	0.19	3.11	0.21	3.54	0.23
4			2.78	0.29	3.01	0.23	3.35	0.16
5			2.38	0.30	2.80	0.28	3.35	0.17

SD—Standard deviation.

## Data Availability

The raw data supporting the conclusions of this article will be made available by the authors on request.
